# Regionale und geschlechtsspezifische Unterschiede bei Appendektomien: Bundesweite kleinräumige Entwicklung der Operationsraten in der Zeitreihe

**DOI:** 10.1007/s00104-022-01628-5

**Published:** 2022-04-07

**Authors:** Lea Leeser, Benno Neukirch, Saskia E. Drösler

**Affiliations:** Fachbereich Gesundheitswesen, Hochschule Niederrhein, Reinarzstraße 49, 47805 Krefeld, Deutschland

**Keywords:** Regionale Unterschiede, Appendektomie, Kleinräumige Analyse, Routinedaten, Inanspruchnahme, Regional variations, Appendectomy, Small-area analysis, Routinely collected health data, Procedures and techniques utilization

## Abstract

**Hintergrund:**

Frühere Analysen kleinräumiger Appendektomieraten zeigen erheblich höhere regionale Unterschiede der Operationshäufigkeiten bei Frauen als bei Männern.

**Ziel:**

Die Arbeit identifiziert valide Messgrößen zur Darstellung regionaler Unterschiede und analysiert geschlechtsspezifische Veränderungen der Appendektomieraten auf Landkreisebene in der Zeitreihe.

**Material und Methoden:**

Datengrundlage sind die der DRG-Statistik entnommenen Appendektomiehäufigkeiten für 2014, 2016 sowie 2018 nach Geschlecht auf Landkreisebene. Die regionalen Unterschiede werden mittels der „systematic component of variation“ (SCV) berechnet und beurteilt. Die SCV ist im Vergleich zu Extrem-Ratio und Variationskoeffizient robuster gegenüber stark schwankender Nennerpopulationen. SCV-Werte über 5 geben einen Hinweis auf hohe Variationen und größer 10 auf sehr hohe Variationen.

**Ergebnisse:**

Bei der männlichen Population lassen sich nur geringe regionale Unterschiede der Operationsraten feststellen, die im Zeitverlauf stabil bleiben (SCV_2014_ = 2,1, SCV_2016_ = 1,8 und SCV_2018_ = 2,0). Bei Frauen hingegen liegt die SCV in den Jahren 2014 sowie 2016 (SCV_2014_ = 6,1, SCV_2016_ = 5,3) über 5 und sinkt 2018 auf 4,5 ab. Darstellungen als Funnel-Plot berücksichtigen höhere Streuungen der Operationsraten in Landkreisen mit niedrigen Einwohnerzahlen.

**Diskussion:**

Bei Frauen ist ein rückläufiger Trend in den Appendektomiehäufigkeiten zu erkennen. Unklar ist, ob dieser Trend auf einer Veränderung der Indikationsstellung oder auf einem geänderten allgemeinen Behandlungsmanagement bei einem Appendizitisverdacht beruht. Durch robuste Variationsmaße und der graphischen Aufbereitung als Funnel-Plots ist es möglich, systematisch bedingte regionale Versorgungsunterschiede von Zufallseffekten zu unterscheiden.

## Abstract (en)

**Background:**

Previous analyses of small-area appendicectomy rates showed significantly higher regional differences in the frequency of operations in women than in men

**Objective:**

This work proposes valid measures to represent regional variations and analyzes gender-specific changes of appendicectomy rates at the county level in the time series.

**Material and methods:**

Appendicectomy frequencies for 2014, 2016 and 2018 by gender and at the county level were taken from the DRG statistics. Regional variations were calculated and assessed using the systematic component of variation (SCV). In comparison to the extreme ratio and coefficient of variation, the SCV is more robust with respect to strongly fluctuating denominator populations. The SCV values greater than 5 indicate high variation and greater than 10 indicate very high variation.

**Results:**

In the male population only minor regional variations in operation rates could be observed, remaining at similar levels over time (SCV_2014_ = 2.1, SCV_2016_ = 1.8, and SCV_2018_ = 2.0). For women the SCV was above 5 in 2014 as well as in 2016 (SCV_2014_ = 6.1, SCV_2016_ = 5.3) and dropped to 4.5 in 2018. Plots as a funnel plot account for higher scatter in surgery rates in counties with low populations.

**Discussion:**

Regarding women, a decreasing trend in regional variation of appendicectomy could be observed. It remains unclear whether this trend reflects a change in the indications or a modified clinical management when appendicitis is suspected. Using robust variation measures and the graphic preparation as funnel plots it is possible to distinguish systematically caused regional differences in care from random effects.

Regionale Unterschiede inkl. der Identifizierung valider Beurteilungsgrößen werden seit einigen Jahrzehnten in der internationalen Fachliteratur sowie in Deutschland thematisiert und publiziert. Differenziert wird hierbei vor allem zwischen gerechtfertigten und ungerechtfertigten Versorgungsunterschieden, wobei letztere zu Defiziten in der Gesundheitsversorgung durch Über‑, Unter- oder Fehlversorgung führen können. Bevölkerungsbezogene Appendektomieraten zeigen in der Vergangenheit besonders für Frauen nennenswerte Versorgungsunterscheide. Da bei dieser Leistung Mehrfacheingriffe ausgeschlossen sind, eignet sie sich besonders gut für Versorgungsanalysen.

## Hintergrund

Regionale Unterschiede der medizinischen Versorgung wurden bereits seit Anfang der 1970er-Jahre durch Wennberg und Gittelsohn untersucht und sind seitdem international Teil der öffentlichen Diskussion. Mit ihrer 1973 veröffentlichten Studie zu Operationshäufigkeiten in 13 Krankenhausbezirken in Vermont identifizierten sie erhebliche regionale Unterschiede beim Ressourceneinsatz, bei der Nutzung von Leistungen sowie Ausgaben für Krankenhausleistungen [20]. Neben den Ursachen für regionale Unterschiede wird die Abbildung der Unterschiedlichkeit als Messgrößen, sog. Variationsmaße, thematisiert. Diese stellen dar, ab wann eine Variation als* auffällig* und wann diese nicht als *zufällig* gilt [4, 9, 13]. International durchgesetzt hat sich die „systematic component of variation“ (SCV), die als robust gegenüber Ausreißern gilt [13]. Mit Beiträgen der OECD sowie der Bertelsmann Stiftung wächst das Interesse an regionalen Variationen auch in Deutschland. Ein Gutachten im Auftrag der Bundesärztekammer thematisiert regionale Variationen und identifiziert auffällige regionale Unterschiede der Appendektomieraten bei Mädchen und Frauen in Deutschland 2012 [10]. Vor allem die Entwicklung der Operationshäufigkeiten sowie der kleinräumigen geschlechtsspezifischen Unterschiede sind Schwerpunkt der vorliegenden Auswertung.

## Fragestellung

Inwiefern lassen sich geschlechtsspezifische Veränderungen der Appendektomiehäufigkeiten in der Zeitreihe feststellen?

Wie entwickeln sich die regionalen Unterschiede auf Ebene der Landkreise (LK)? Sind weiterhin die Unterschiede der Operationsraten bei den Frauen größer als bei den Männern?

Ist eine Beschreibung der regionalen Unterschiede mittels SCV und Funnel-Plots sachgerechter als ein alleiniger Vergleich von Operationsraten?

## Methoden

Datengrundlage sind die durch das Fachreferat H1 – Gesundheit des Statistischen Bundesamtes bereitgestellten Abrechnungsdaten der Krankenhäuser nach § 21 Krankenhausentgeltgesetz (DRG-Daten). Für die vorliegende Analyse der Appendektomiehäufigkeiten werden die Operationen- und Prozedurencodes (OPS-Codes der Gruppe 5‑470.-, Appendektomie als eigenständiger Eingriff) der Datenjahre 2014, 2016 und 2018 verwendet. Die Datenlieferung erfolgt für den Leistungsbereich Appendektomie geschlechtergetrennt nach 21 Altersklassen (0–1, 1–5, 5–10, 10–15, 15–20, 20–25, 25–30, 30–35, 35–40, 40–45, 45–50, 50–55, 55–60, 60–65, 65–70, 70–75, 75–80, 80–85, 85–90, 90–95, 95 und älter) auf regionaler Ebene für alle rund 400 deutschen Landkreise und kreisfreien Städte sowie bundesweit. Die Zuordnung der Appendektomie zu den jeweiligen Landkreisen und kreisfreien Städten erfolgt nach Wohnort des Patienten/der Patientin und nicht nach Standort des Krankenhauses. Mittels der Bevölkerungsdaten der Regionaldatenbank des Statistischen Bundesamtes konnten bevölkerungsbezogene geschlechtsspezifische Operationsraten nach allen deutschen Landkreisen und kreisfreien Städten ermittelt werden [5–7]. Auf Kreisebene wird eine indirekte Altersstandardisierung durchgeführt, die im Gegensatz zur direkten Standardisierung bei niedrigen Fallzahlen Anwendung findet und als Voraussetzung bei der Berechnung der SCV gilt [13, 14]. Bei der indirekten Altersstandardisierung werden für jede Beobachtungseinheit (LK) die Anzahlen der beobachteten Appendektomiefälle durch die Anzahl der nach Altersstruktur der Bevölkerung eines LK erwarteten Appendektomiefälle dividiert (O/E-Ratio). Die erwarteten Fallzahlen pro Landkreis ergeben sich aus der Summe der gewichteten Fallzahlen pro Altersklasse, wobei der Wichtungsfaktor die gesamtdeutsche Operationsrate der jeweiligen Altersklasse ist. Auf die bevölkerungsbezogene Standardrate pro LK wird anhand des Produkts aus Gesamtrate und O/E-Ratio zurückgerechnet. Zusätzlich wurden ebenfalls die indirekt standardisierten Raten unter Verwendung der Altersstruktur von 2014 für alle drei Datenjahre berechnet, um eventuelle Effekte der geänderten Altersstruktur zu bereinigen.

Zur Berechnung der regionalen Variation auf Landkreisebene wird die SCV verwendet. Im Gegensatz zu den gängigen Variationsmaßen „coefficient of variation“ (CV) und dem „extremal quotient“ (EQ), welche Variationen auf Basis der beobachteten Werte ermitteln, berechnet die SCV Variationen auf Grundlage der O/E-Ratio (Verhältnis der beobachteten zu den erwarteten Fallzahlen). Ibanez und Kollegen beschreiben die SCV im Gegensatz zu anderen Variationsmaßen als besonders geeignet, da sie bei bevölkerungsbedingten Unterschieden in der Operationshäufigkeit stabil bleibt [13]. Auch McPherson, der dieses Verfahren erstmalig beschreibt, bestätigt, dass die SCV gegenüber den Variationsmaßen CV und EQ den Vorteil hat, dass demographische Aspekte der Bevölkerungsstruktur Berücksichtigung finden und vor allem robust gegenüber Ausreißern sind [16]. Ibanez und Kollegen stellen weiterhin fest, dass es aufgrund schwankender Operationsraten sinnvoll ist, nicht nur die beobachteten Werte, sondern ebenfalls die erwarteten Werte zu berücksichtigen. So wird die SCV nicht durch die Prozedurenrate beeinflusst und ist in der Lage, bei stark unterschiedlichen Operationshäufigkeiten stabil zu bleiben [13].

Zur Berechnung der SCV wird die folgende Formel verwendet:

$$\text{SCV}=\frac{1}{I}\left(\sum \frac{\left(y_{i}-e_{i}\right)^{2}}{{e}_{i}^{2}}-\sum \frac{1}{e_{i}}\right)\times 100$$

wobei y_i_ der beobachteten Rate, e_i_ der erwarteten Rate und I der Anzahl der betrachteten Regionen entsprechen.

Birkmeyer und Kollegen bestätigen die SCV als robusten und aussagekräftigen Messwert für regionale Variationen, nach Alters- und Geschlechterstandardisierung und benennen in Anlehnung an Appleby und Kollegen SCV-Werte größer 5 als auffällig bzw. hohe Variationen und SCV-Werte größer als 10 als sehr auffällig bzw. sehr hohe Variationen [2, 4].

Mithilfe von Funnel-Plots wird graphisch dargestellt, ob Werte eines Kreises innerhalb („common cause variation“) der Kontrollgrenzen von 2 (95 %) oder 3 (98,8 %) Standardabweichungen (SD) liegen und durch den Zufall erklärt werden können oder ob der Wert des Kreises außerhalb („special cause variation“) liegt und als auffällig interpretiert werden kann [18]. Mittels einer kartographischen Darstellung werden die Landkreise und kreisfreien Städte, die außerhalb der 3. SD liegen, kenntlich gemacht [10]. Die Funnel-Plots werden mit der Eingabemaske der Association of Public Health Observatories erstellt [1], die Landkarten mit dem Programm RegioGraph 2020 der GfK GeoMarketintg GmbH.

## Ergebnisse

**Figure 1:**
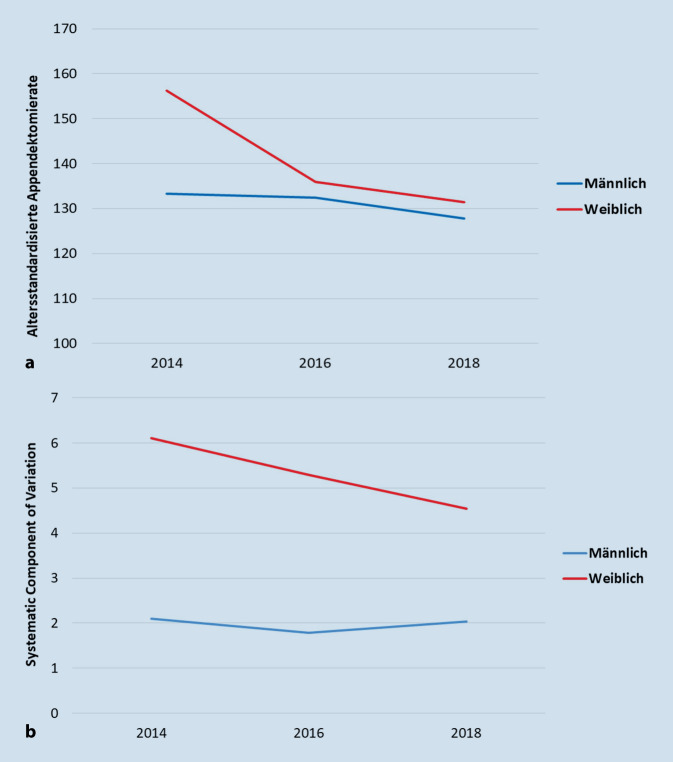

Die absolute Zahl der Appendektomien sinkt im Zeitverlauf von 116.348 Appendektomien (männlich: 53.399, weiblich: 62.949) im Jahr 2014 auf 110.953 Appendektomien (männlich 53.434, weiblich: 57.519) im Jahr 2016 und auf 108.124 Appendektomien (männlich: 52.656, weiblich: 55.468) im Jahr 2018. Insgesamt sinken die Mediane der Appendektomieraten über alle rund 400 LK von 2014 bis 2018 erheblich, wobei die Reduzierung bei Mädchen und Frauen deutlicher zu erkennen ist als bei Jungen und Männern. Die Mediane der männlichen Population bleiben von 2014 bis 2016 ca. auf gleicher Höhe und sinken nur marginal von 133,3 auf 132,4 pro 100.000 Einwohnerinnen und Einwohner (EW). Nach 2016 ist eine Reduzierung auf 127,8 Appendektomien pro 100.000 EW zu erkennen. Auffällig ist zusätzlich, dass die Operationsrate der Mädchen und Frauen im Jahr 2014 rund 17 % höher ist als die der Jungen und Männer. Im Jahr 2018 beträgt der Unterschied zwischen den Geschlechtern nur noch ca. 3 % (Abb. 1a).

Auch auf kleinräumiger regionaler Ebene zeigen sich geschlechtsspezifische Unterschiede: In der Zeitreihe ist ein Rückgang der Unterschiedlichkeit, ausgedrückt durch die SCV, zwischen beiden Geschlechtern erkennbar. Bei Mädchen und Frauen verändert sich die SCV von 6,1 auf eine SCV von unter 5. Bei Jungen und Männern ist zunächst ein Rückgang von 2,2 (2014) auf 1,9 (2016) zu beobachten, woraufhin ein leichter Anstieg auf ca. 2,0 (2018) erfolgt. Trotz des deutlicheren Rückgangs bei der weiblichen Population liegen die SCV-Werte über alle Jahre hinweg über denen der männlichen Population (Abb. 1b).

**Figure 2:**
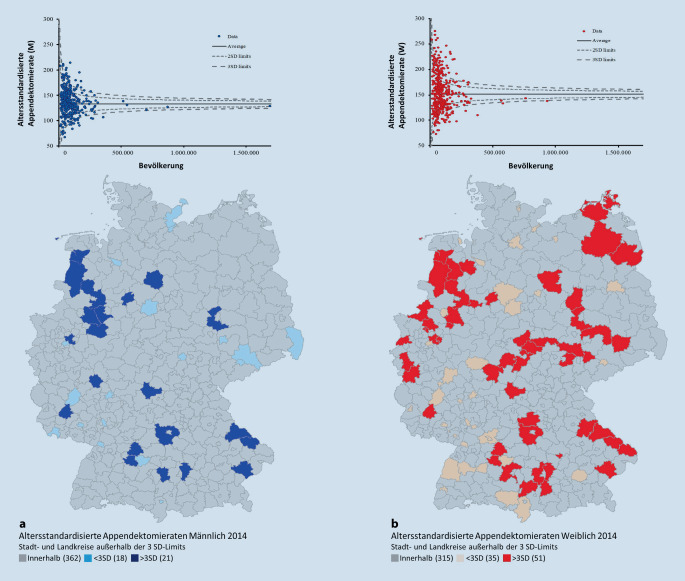

Die Funnel-Plots (Abb. 2, 3 und 4) der Appendektomieraten auf Landkreisebene zeigen in allen Jahren Über- sowie Unterschreitungen der Kontrollgrenzen als Hinweis auf eine systematische Unterschiedlichkeit der Operationsraten. Über alle Jahre hinweg ist eine deutlich höhere Streubreite der Operationsraten in LK mit geringeren Bevölkerungsanzahlen zu erkennen. Für die weibliche Population finden sich deutlich mehr Landkreise mit Operationsraten außerhalb der 3. Standardabweichung als bei den Männern.

**Figure 3:**
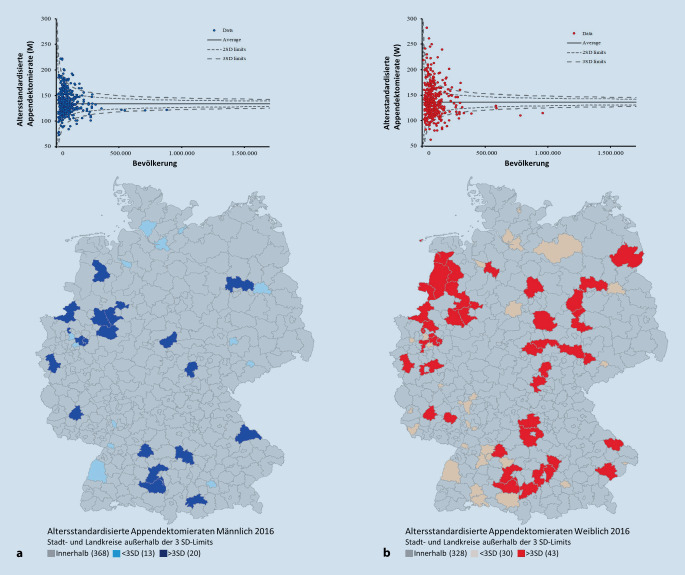

**Figure 4:**
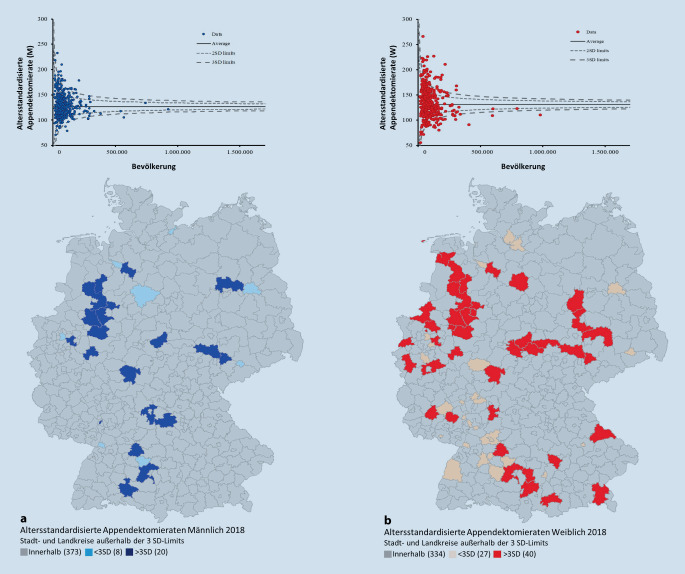

In Tab. 1 sind für beide Geschlechter die Anzahlen der LK mit auffälligen Operationsraten unter- bzw. oberhalb der Funnel-Kontrollgrenzen dargestellt. Im Jahr 2014 lassen sich bei der männlichen Population rund 18 LK unterhalb der Kontrollgrenzen und 21 LK oberhalb der Kontrollgrenzen identifizieren. Bei Mädchen und Frauen liegen 35 LK unterhalb und 51 LK oberhalb der Kontrollgrenzen. So liegen ca. doppelt so viele LK bei der weiblichen Population unterhalb der Kontrollgrenzen und fast 2,5-mal so viele LK oberhalb der Kontrollgrenzen im Vergleich zur männlichen Population (Abb. 2). In der Zeitreihe verringern sich zwar die jeweiligen Anzahlen, dennoch liegen im Jahr 2018 bei Mädchen und Frauen im Vergleich zur männlichen Population weiterhin mehr als doppelt so viele LK außerhalb der Kontrollgrenzen (Abb. 4).

**Table 1:** 

		Männlich	Weiblich
2014	< 3 SD	18	35
	> 3 SD	21	51
2016	< 3 SD	13	30
	> 3 SD	20	43
2018	< 3 SD	8	27
	> 3 SD	20	40

*<* *3 SD* unterhalb der Funnel-Kontrollgrenzen; *>* *3 SD* oberhalb der Funnel-Kontrollgrenzen

In den Ländern Bayern, Thüringen, aber auch Nordrhein-Westfalen und Sachsen-Anhalt findet sich für die weibliche Population eine Häufung der auf hohen Raten basierenden Ausreißern, die im Funnel-Plot oberhalb der Kontrollgrenzen liegen und in den Landkarten dunkelrot markiert sind. Geringe Raten – LK unterhalb der Kontrollgrenzen – sind vermehrt in Baden-Württemberg sowie Bremen zu identifizieren.

Der lineare Zusammenhang der Operationsraten zwischen beiden Geschlechtern wird anhand einer Korrelationsanalyse überprüft. Der Korrelationskoeffizient nach Pearson sinkt zwar von 2014 bis 2018 von 0,71 (*p* = 0,01) auf 0,66 (*p* = 0,01), zeigt jedoch über alle Jahre hinweg einen deutlichen positiven Zusammenhang zwischen den Appendektomieraten bei Frauen und Männern.

Die Ergebnisse der auf die Bevölkerungsanzahlen des Jahrs 2014 standardisierten Operationsraten aus den Jahren 2016 und 2018 zeigen keine wesentlichen Unterschiede und werden deshalb nicht weiter ausgeführt.

## Diskussion

Die Zeitreihe zeigt sinkende Eingriffshäufigkeiten. Zwischen 2012 [10] und 2018 fällt der Median der altersstandardisierten Appendektomierate von 156,0 auf 129,9 Appendektomien pro 100.000 EW. Bestätigt werden zusätzlich die geschlechterspezifischen Unterschiede der Appendektomieraten, wobei festzustellen ist, dass auch der Unterschied der Eingriffshäufigkeit zwischen beiden Geschlechtern sinkt.

Ähnliche Entwicklungen zeigen sich für die regionale Unterschiedlichkeit, ausgedrückt durch das Variationsmaß SCV. Sie liegt bei Jungen und Männern um den Wert von 2,0, was als unauffällig bewertet wird. Bei den Mädchen und Frauen hingegen ist eine abnehmende Tendenz zu beobachten, von 5,5 [9] in 2012 auf 4,5 in 2018. Dennoch liegt sie weiterhin deutlich über dem Wert der Jungen und Männer.

Wennberg beschreibt, dass bei der Versorgung der akuten Appendizitis die behandelnde Ärztin oder der behandelnde Arzt in den meisten Fällen die Entscheidung für oder gegen einen operativen Eingriff vollständig alleine trifft – ohne Einbeziehung der Patientin oder des Patienten – und somit die persönliche Erfahrung und der Behandlungsstil ausschlaggebend für die Therapie sind. Somit kann in Einzelfällen eine Fehlversorgung nicht ausgeschlossen werden [21].

Weiterhin geht die Appendizitis häufig mit einer nicht einheitlichen Symptomatik einher, die die Problematik zahlreicher Differenzialdiagnosen mit sich bringt. Möglicherweise indizieren einige Ärztinnen oder Ärzte, trotz unsicherer Diagnose, schneller eine Appendektomie als andere. Für die vorliegende Untersuchung ist allerdings zu beachten, dass nicht jede Appendektomie mit einer Appendizitis assoziiert ist. Besonders bei Frauen gibt es eine hohe Anzahl an Differenzialdiagnosen, die fälschlich auf eine Appendizitis hindeuten und somit eine Operation nach sich ziehen [12]. Die höheren altersstandardisierten Appendektomieraten in den Jahren 2014, 2016 und 2018 bei Mädchen und Frauen im Vergleich zu Jungen und Männern stützen diese Vermutung [8].

Es stellt sich die Frage, inwieweit eine eindeutige Indikationsstellung durch einen standardisierten Behandlungspfad oder eine Leitlinie unterstützt wird und damit vor allem die bei Mädchen und Frauen beobachteten regionalen Unterschiede reduziert werden könnten. So würde das Vorgehen beim Verdacht auf eine akute Appendizitis vereinheitlicht und der Entscheidungsspielraum für behandelnde Ärztinnen und Ärzte eingeschränkt. Besonders bei Frauen, aber auch bei Hochbetagten sowie Kindern wird die Diagnostik als schwierig beschrieben, da diese eher eine unspezifische Symptomatik und eine Vielzahl von Differenzialdiagnosen aufweisen können [11]. Das weibliche Geschlecht zeigt sich zudem als Risikofaktor für eine Nonappendizitisappendektomie, die gemäß Bhangu bei Frauen zwischen 16 und 45 Jahren um 28 % erhöht ist [3].

Der Rückgang der Appendektomieraten lässt sich möglicherweise auch durch den aktuellen Trend der Antibiotikatherapie als Behandlung bei ausgewählten Patientinnen und Patienten erklären. So zeigen Stöß und Kollegen in einer aktuellen Untersuchung den Rückgang der Appendektomien bei einer akuten Appenditzitis mit einer relativen Reduktion von 11,5 % im Zeitraum von 2010 bis 2017, bei Zunahme der komplizierten Fälle mit perforierter Appendizitis [19]. Es ist zu erkennen, dass die absoluten Fallzahlen von Patientinnen und Patienten mit einer Hauptdiagnose „akute Appendizitis“ (K35) in den Jahren 2014 bis 2018 nur leicht (2014: 100.214; 2016: 98.889; 2018: 98.075) sinken [8]. Anhand der hier analysierten Daten, die sich auf reine Operationshäufigkeiten beschränken, lässt sich jedoch nicht abschließend klären, wieso die Appendektomieraten im zeitlichen Verlauf abnehmen.

Unter Berücksichtigung der Bevölkerungsanzahlen (x-Achse) werden die altersstandardisierten Appendektomieraten (y-Achse) als Funnel-Plot aufgetragen. Mithilfe dieser Methode werden auffällig hohe und niedrige Raten identifiziert und die korrespondierenden LK im Flächenkartogramm farblich kenntlich gemacht. Bemerkenswert ist hierbei, dass besonders bei den Operationsraten der Frauen einige wenige Landkreise mit auffällig niedrigen Werten an Grenzen zum Ausland liegen und somit möglicherweise ein eingeschränktes Einzugsgebiet haben. Dies ist bei der Untersuchung von Ausreißern in LK, die ans Ausland grenzen, zu beachten. Weiterhin zeigt die Funnel-Plot-Methode eindrucksvoll, dass bei Landkreisen mit niedrigen Bevölkerungszahlen die zufällig bedingte Unterschiedlichkeit der Operationsraten sehr hoch sein kann und nicht als Auffälligkeit zu bewerten ist.

Beispielsweise konnte für den LK Cochem-Zell eine hohe Appendektomierate von 170,14 pro 100.000 EW für Jungen und Männer und von 171,3 pro 100.000 EW für Mädchen und Frauen identifiziert werden. Die Bevölkerungszahl liegt dort für das Jahr 2018 bei 30.841 männliche Population sowie 30.746 weibliche Population. Aufgrund der niedrigen Bevölkerungszahl ist die Unterschiedlichkeit der Operationsraten nicht als auffällig zu bewerten. Die Appendektomierate für Jungen und Männer liegt in Pirmasens bei 93,31 pro 100.000 EW bei einer männlichen Bevölkerung von 40.403. Trotz der im Vergleich zu anderen LK geringen Appendektomierate gilt die Unterschiedlichkeit der Appendketomierate in Pirmasens als nicht auffällig. Bei Mädchen und Frauen liegt der LK Lindau mit 97,3 pro 100.000 EW bei einer weiblichen Bevölkerung von 41.384 innerhalb der Kontrollgrenzen und gilt ebenfalls als nicht auffällig.

Zudem konnte durch eine Korrelationsanalyse gezeigt werden, dass in den LK, wo viele Mädchen und Frauen operiert werden, auch viele Jungen und Männer appendektomiert werden. Dies stützt die Vermutung, dass vor allem der individuelle Behandlungsstil und Erfahrungen der Ärztinnen und Ärzte ausschlaggebend für die Therapiemethode ist [21].

## Limitationen

Inwieweit sich Abrechnungsdaten für Versorgungsanalysen eignen, wird anhaltend diskutiert. Als problematisch werden hierbei beispielsweise die Kodierqualität oder ein eingeschränkter Informationsgehalt erachtet, da klinische Details fehlen könnten [15, 22]. Durch Kodierrichtlinien, Fallprüfungen sowie Plausibilitätsprüfungen der InEK-Datenstelle wird jedoch versucht, die externe Validität zu erhöhen [17]. Aus diesem Grund und vor allem durch die lückenlose Aufführung der Daten beim Statistischen Bundesamt seit 2005 sind die DRG-Daten für Analysen, die sich vor allem mit Leistungshäufigkeiten im Krankenhaus befassen, geeignet. Hinzu kommt, dass sich der Eingriff Appendektomie eindeutig nach OPS verschlüsseln lässt. Dennoch sind Abweichungen zur tatsächlichen Situation der Versorgung nicht vollständig auszuschließen.

Bei den vorliegenden Daten wird die Zuordnung der Appendektomie zu den jeweiligen Landkreisen und kreisfreien Städten nach dem Wohnort der Patientin und des Patienten und nicht nach dem Standort des Krankenhauses durchgeführt. So sind Verzerrungen nicht ausgeschlossen, wenn eine Patientin oder ein Patient ein Krankenhaus aufsucht, das weiter entfernt vom Wohnort liegt. Es ist nicht möglich, auf Landkreisebene krankenhausbezogen Operationshäufigkeiten beim Statistischen Bundesamt anzufragen [10].

Kleinräumige Versorgungsanalysen auf der Ebene der rund 400 deutschen LK bieten für operative Disziplinen, die gelegentlich mit Fragen nach Überversorgung konfrontiert werden, einen detaillierten Einblick in das regionale Versorgungsgeschehen. Bei auf dieser Ebene durchgeführten Vergleichen ist die Abhängigkeit der zufälligen Streuung der bevölkerungsbezogenen Operationsrate von der Bevölkerungsanzahl in der beobachteten Versorgungsregion zu beachten. Funnel-Plots können hierbei hilfreich sein. Die Appendektomie ist für eine derartige Analyse prädestiniert, da sie ausschließlich stationär und pro Fall nur einmalig durchgeführt wird. Hier gibt es bei der Versorgung von Jungen und Männern keine gravierenden regionalen Unterschiede, bei Mädchen und Frauen sind die regionalen Operationsunterschiede deutlich rückläufig.

## Fazit für die Praxis

- Die Beurteilung regionaler Versorgungsunterschiede auf Basis der Inanspruchnahme sollte nicht alleinig auf bevölkerungsbezogenen Versorgungshäufigkeiten beruhen, sondern auf robusten Variationsmaßen und dabei geschlechtsspezifische Unterschiede berücksichtigen.
- Sowohl bei der männlichen als auch bei der weiblichen Population ist ein sinkender Trend der regionalen Unterschiede auf der Ebene der Landkreise zu erkennen.
- Sinkende Appendektomieraten können auf sinkende Fallzahlen mit akuter Appendizitis hindeuten oder auf eine vermehrte nichtoperative Therapie.
- Der statistisch positive Zusammenhang zwischen den Operationsraten der Männer und der Frauen belegt, dass es auf der Ebene der Landkreise keine geschlechtsspezifischen Unterschiede für das Therapieregime gibt.

## Abkürzungen

CV: Coefficient of variation
EQ: Extremal quotient
EW: Einwohnerinnen und Einwohner
LK: Landkreis/e
SCV: Systematic component of variation
SD: Standardabweichung
